# Supplementary material for: Complexome profiling of the Chlamydomonas psb28 mutant reveals TEF5 as an early PSII assembly factor
Source: Plant Cell. 2025 Mar 17;37(6):koaf055. doi: 10.1093/plcell/koaf055 (PMC12164590; doi:10.1093/plcell/koaf055)
Supplement: koaf055_Supplementary_Data [file koaf055_supplementary_data.zip › psb28_interactive.zip!/plots/intensity/qualityPlots/peptides/HCD1_Cre16.g695050.t1.2.html]

### Cre16.g695050.t1.2

Trivial name: HCD1  
SeqCoverage [median]: 31.60  
Mass: 76.54 kDa  
  
 

| Sequence | MissedCleavage | Mass [Da] | wtIntensity | muIntensity | Score |
| --- | --- | --- | --- | --- | --- |
| ADMVIEAVIEDIPLKQK | 1 | 1911.04 | 1410.35 | 0.00 | 84.56 |
| AFAAAAALDTHK | 0 | 1185.61 | 0.00 | 1219.02 | 85.85 |
| ALALDIAAGR | 0 | 969.56 | 2369.49 | 7996.26 | 113.71 |
| ALVHIFFAQR | 0 | 1200.68 | 0.00 | 974.20 | 81.30 |
| APQPEAQYR | 0 | 1058.51 | 558.24 | 0.00 | 42.11 |
| EVSQQFLDGGMAR | 0 | 1436.67 | 0.00 | 576.70 | 139.31 |
| FGMPMGPFR | 0 | 1038.48 | 0.00 | 474.82 | 49.06 |
| GAMSQAAADAALGR | 0 | 1288.62 | 1841.38 | 3879.14 | 184.34 |
| GGQEGLAAEGR | 0 | 1043.50 | 3005.79 | 0.00 | 93.10 |
| GVTDVPGLKPR | 1 | 1137.65 | 0.00 | 363.13 | 36.42 |
| KASPDPEGLAPLLEGSR | 1 | 1735.91 | 572.53 | 0.00 | 36.11 |
| KGAMSQAAADAALGR | 1 | 1416.71 | 307.33 | 0.00 | 77.42 |
| KLSAGPPAAAKL | 2 | 1122.68 | 263.40 | 0.00 | 70.73 |
| LGLVDVLAPDAAGLMPAAR | 0 | 1849.01 | 821.06 | 0.00 | 63.83 |
| LIPALNEAK | 0 | 967.57 | 779.22 | 922.40 | 61.96 |
| LIPALNEAKR | 1 | 1123.67 | 1169.45 | 0.00 | 42.40 |
| TDKLPPLGEALALLDMAR | 1 | 1923.05 | 1315.51 | 0.00 | 84.92 |
| VDAAVAGR | 0 | 757.41 | 1073.99 | 1801.45 | 116.54 |
| VSGALDYAGFAR | 0 | 1225.61 | 3974.09 | 10107.40 | 121.50 |
|  |  |  | 19461.8 | 28314.5 |  |
